# Investigating the Effects of Heated Tobacco Products on Periodontal Healing: Insights From In Vivo and In Vitro Experiments

**DOI:** 10.7759/cureus.80733

**Published:** 2025-03-17

**Authors:** Kengo Doya, Kentaro Imamura, Shinta Mori, Saki Nakane-Koyachi, Eitoyo Kokubu, Kazuyuki Ishihara, Atsushi Saito

**Affiliations:** 1 Periodontology, Tokyo Dental College, Tokyo, JPN; 2 Oral Health Science Center, Tokyo Dental College, Tokyo, JPN; 3 Microbiology, Tokyo Dental College, Tokyo, JPN

**Keywords:** alveolar bone, cigarette smoke, gingival fibroblasts, heated tobacco products, periodontal tissue, periodontitis

## Abstract

This study aimed to investigate the effects of heated tobacco product extract (HTPE) on periodontal healing. HTPE and conventional cigarette smoke extract (CSE) were prepared following a puff protocol. C57BL/6 mice were divided into HTPE, CSE, and control groups. Each solution was intraperitoneally administered for three days. Seven days after ligature placement in the maxillary second molar, alveolar bone resorption was assessed. Bone levels after ligature removal were also assessed. In vitro, human gingival fibroblasts-1 (HGF-1) were cultured with HTPE or CSE for 24 hours. Cell proliferation, migration, and morphology were evaluated, and expressions of genes related to cell migration were also assessed. HTPE and CSE induced significantly greater bone resorption compared with the control, and bone recovery in both groups was reduced. While HTPE had no significant impact on the proliferation of HGF-1, CSE suppressed it. Both HTPE and CSE significantly inhibited cell migration. In the HTPE group, actin filaments in HGF-1 were more pronounced compared with the control, and matrix metalloproteinase-1 (MMP-1) and matrix metalloproteinase-3 (MMP-3) gene expressions were significantly increased. The suppressed cell migration may be attributed in part to the regulation of extracellular matrix remodeling. The results of this study suggest that HTPE impairs periodontal healing, although the extent of this effect may differ from that of CSE.

## Introduction

Smoking is an important environmental risk factor for periodontitis. Smokers have approximately three times the probability of developing periodontal disease compared to non-smokers [1]. Moreover, the effectiveness of periodontal treatment has been demonstrated to be lower in smokers compared to non-smokers.

Smoked tobacco products contain more than 7000 chemical substances, including at least 250 known to be toxic or to cause cancer [2]. Among these, nicotine, tar, and carbon monoxide are considered the most harmful substances. Tobacco use is detrimental to the vasculature as it induces microvascular dysfunction, thereby exerting a negative impact on periodontal disease [3]. Additionally, the various toxic compounds of cigarette smoke may trigger pro- and suppressive immunoinflammatory effects on periodontal tissues [4]. Nicotine inhibits the proliferation and adhesion of cells in periodontal tissues [5]. It also promotes the production of inflammatory mediators.

The adverse effects of smoking/vaping on wound healing are widely recognized. Such effects are induced by toxic substances such as nicotine, carbon monoxide, and hydrogen cyanide [6]. In periodontal treatment, especially in periodontal surgery, impaired healing in smokers has been reported [7]. Previously, we reported that cigarette smoke condensate inhibited the migration of human gingival epithelial cells [8].

In recent years, new types of tobacco, such as heat-not-burn/heated tobacco products (HTPs) and electronic cigarettes, have emerged. HTPs generate nicotine-containing aerosols by heating tobacco leaves instead of burning them. HTPs have three primary components: a tobacco stick, a battery-powered tobacco heating holder, and a charger. The principal distinction between conventional cigarettes (CC) and HTPs is that while CC is burned at temperatures exceeding 600°C, HTPs heat the tobacco stick to below 350°C [9]. Heating the tobacco stick generates an aerosol primarily composed of water, glycerol, and nicotine, with other harmful and potentially harmful constituents. It was reported that HTPs contain lower levels of harmful constituents, such as nicotine and tar in vapor/aerosol compared to CC [10].

Despite the potential reduction of harmful substances contained in HTPs, previous studies have reported their systemic effects. The use of HTPs has been shown to impair myocardial systolic and diastolic function similarly to CC [11]. It was reported that short-term use of HTPs by healthy young adults impaired vascular function and increased the formation of arterial stiffness and platelet thrombosis [12]. Use of HTPs may increase risks of coronavirus infection and severity of coronavirus disease 2019 (COVID-19) [13].

In a population of healthy young adults, the use of HTPs inhibited the local inflammatory response in the oral cavity [14]. A systematic review and meta-analysis suggested that HTPs have detrimental effects on periodontal and periimplant parameters, and laboratory tests showed the presence of carcinogenic and inflammatory biomarkers [15]. However, the detailed influence of HTPs on periodontal tissues remains unclear.

To our knowledge, no in vivo studies have investigated how HTPs affect periodontal tissues. Given the potential link between HTPs and periodontal disease, waiting for epidemiological evidence would be imprudent. Therefore, we opted to conduct basic experiments to examine these effects at the tissue and cellular levels. The purpose of this study was to investigate the effects of HTP extract (HTPE) on periodontal tissue by in vivo and in vitro experiments.

The part of this article was previously presented as a meeting abstract at the 110th Annual Meeting of the American Academy of Periodontology in collaboration with the Japanese Society of Periodontology and the Japanese Academy of Clinical Periodontology on November 2, 2024.

## Materials and methods

Test materials

HTPE was produced from HTP (device/tobacco stick: IQOS 3DUO/Marlboro Heat Sticks Regular; Philip Morris Japan, Tokyo, Japan). Cigarette smoke extract (CSE) was obtained from a CC (Marlboro Red Soft Pack; Philip Morris Japan).

Preparation of HTPE and CSE

**Figure 1:**
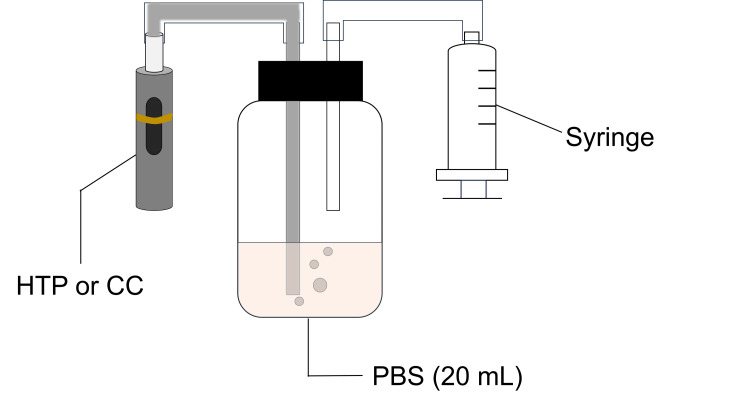
Preparation of heated tobacco product extract (HTPE) and cigarette smoke extract (CSE). HTP: heated tobacco product; CC: conventional cigarettes; PBS: phosphate-buffered saline Credit: Image created by the authors.

The following method was used to generate HTPE and CSE [16]: a four-second puff every 30 seconds, with a volume of 55 mL/puff (Figure 1).

The number of puffs varied depending on the device: eight puffs in 220 seconds for CC and 10 puffs in 318 seconds for HTP. The preparation of the smoke extracts was carried out as previously described [16]. Using a syringe, smoke or aerosol was passed through 20 mL of phosphate-buffered saline (PBS, pH 7.4), which was then filtered through a 0.22 µm filter (Merck Millipore, Billerica, MA, USA). A pilot experiment was carried out to identify the concentration of the extracts which exerted no cytotoxic effect on human gingival fibroblasts-1 (HGF-1). The extracts were prepared from one or two units and labeled as 100% or 200% preparations. HTPE and CSE were used either as undiluted or serially diluted.

Analysis of nicotine concentration in HTPE and CSE

The samples (100% HTPE and CSE preparations) were kept at -80°C and sent to a commercial laboratory (Shimadzu Techno- Research, Kyoto, Japan). The nicotine concentrations were quantified by using high-performance liquid chromatography (HPLC) equipped with CERI L-Column2 ODS 5 µm (150 mm × 4.6 mm i.d.; Chemco, Osaka, Japan) and SPD-M20A detector (Shimadzu Corporation, Kyoto, Japan).

Animals

Eight- to nine-week-old male mice were utilized (C57BL/6J; 20 to 30 g; Sankyo Labo Service, Tokyo, Japan). Each mouse was housed separately in its cage within a climate-regulated environment, with unrestricted access to standard laboratory feed and drinking water. This study was conducted according to the Tokyo Dental College Guidelines for the Animal Experiment (approval no. 242206) and the ARRIVE (Animal Research: Reporting of In Vivo Experiments) guidelines (https://www.nc3rs.org.uk/arrive-guidelines).

In vivo protocol and micro-computed tomography (micro-CT)

**Figure 2:**
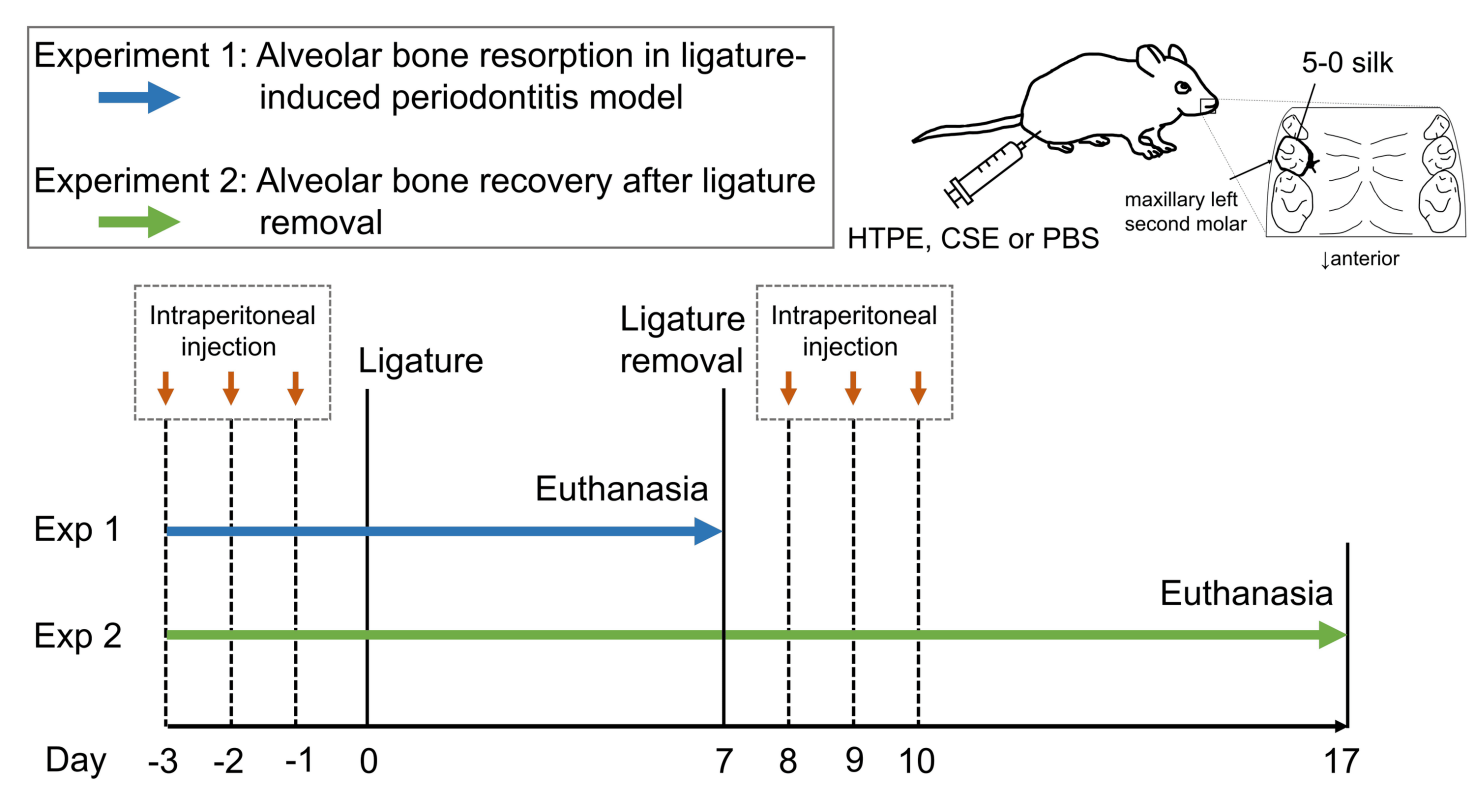
In vivo protocol. HTPE: heated tobacco product extract; CSE: cigarette smoke extract; PBS: phosphate-buffered saline Credit: Image created by the authors.

The in vivo protocol is shown in Figure 2.

Experiment 1: Alveolar Bone Resorption in Ligature-Induced Periodontitis Model

Three groups were created using 32 mice: one group received PBS (control), and two groups were injected with 20 mL/kg of HTPE or CSE. This represents a nicotine dose of approximately 0.83 mg/kg, which corresponds to the daily consumption of 10 cigarettes [17]. Mice received daily intraperitoneal injections of PBS, CSE, or HTPE for three days [18]. To induce periodontitis in mice, mice were anesthetized by intraperitoneal injection of midazolam (2 mg/kg), medetomidine (0.15 mg/kg), and butorphanol (2.5 mg/kg). A ligature (5-0 silk) was tied around the maxillary left second molar [19,20] on day 0. The ligature was gently tied in a manner to avoid damage to the periodontal tissue. In each mouse, the contralateral tooth was not ligated as a control. During the experimental period, the ligatures remained intact in all mice.

On day 7, the mice were anesthetized and euthanized. The maxilla were retrieved and analyzed by a micro-CT system (R-mCT; Rigaku, Tokyo, Japan) with the settings on field of view 10 × 10 mm, tube voltage 90 kV, and tube current 88 μA. The micro-CT data were visualized by TRI/3D-BON software (Ratoc System Engineering, Tokyo, Japan) and measured using ImageJ (National Institute of Health, Bethesda, MD; https://imagej.net/ij/). The bone resorption was assessed by the method described previously [19,20]. Measurements were taken at six sites along the palatal surface of the maxillae to determine the distance between the cemento-enamel junction (CEJ) and the alveolar bone crest (ABC). Each measurement was summed up to calculate the total CEJ-ABC distance. Bone resorption was calculated using the following formula: the difference between the total CEJ-ABC measurements from the ligated side and those from the non-ligated control side within each mouse.

Experiment 2: Alveolar Bone Recovery After Ligature Removal

Twenty-one mice were divided into PBS (control), CSE and HTPE groups. Mice received intraperitoneal injections of each solution for three days (days -3, -2, and -1), and then a ligature was placed around the maxillary left second molar on day 0. The ligature was removed from the mice on day 7. The mice continued to receive PBS, CSE or HTPE injections on days 8, 9, and 10 [18]. On day 17, the mice were euthanized under anesthesia, and the maxillae were used for micro-CT analysis.

Cell culture

The human gingival fibroblast cell line HGF-1 (ATCC CRL-2014; American Type Culture Collection, Manassas, VA, USA) was used for in vitro experiments. Cells were cultured in Dulbecco's Modified Eagle's Medium (DMEM; ATCC 30-2002, American Type Culture Collection) supplemented with 10% fetal bovine serum (Gibco, Invitrogen, Carlsbad, CA, USA), 100 U/mL penicillin, and 100 μg/mL streptomycin (FUJIFILM Wako Pure Chemical Corporation, Osaka, Japan) at 37°C in a humidified atmosphere containing 5% CO_2_ until confluence or subconfluence. The cells were passaged by trypsinization (0.25% trypsin and 0.02% ethylenediaminetetraacetic acid, or EDTA), counted in a cell counter (Thermo Fisher Scientific, Waltham, MA, USA) and plated as described below. All experiments were performed between the 2-9 subcultures.

Cell viability/proliferation assay

HGF-1 were seeded (0.5 × 10^4^ cells/well) on microplates (96-well; Falcon, Corning, NY, USA) and incubated for 24 hours at 37°C in 5% CO_2_. The culture medium was replaced with 100 μL of the extracts (HTPE; 6.25-200% or CSE; 3.12-100%). A fresh culture medium was added to the control. They were incubated for a further 24 hours. Then, cell viability was evaluated using the WST-8 (Cell Counting Kit-8; Dojindo Laboratories, Kumamoto, Japan), according to the manufacturer's protocol. The obtained supernatants were assessed for absorbance at 450 nm.

Wound healing assay

Confluent HGF-1 monolayers were incubated with DMEM, HTPE, or CSE with the concentration range that included salivary nicotine concentrations of smokers [21]. After 24 hours, three artificial wounds per well were made using a pipette tip [22]. The cells were washed quickly to remove the floating detached cells. The wound area was monitored for up to 24 hours using a phase-contrast microscope (Eclipse TE-DH; Nikon, Tokyo, Japan) equipped with a digital micro-camera system (FDX-35; Nikon). The cells were maintained in a tissue culture incubator between image acquisitions. At each time point, the wound area was measured using ImageJ. The percent wound closure was calculated using the equation:

\[ \text{Percent wound closure} = \frac{(\text{Initial wound area}) - (\text{Wound area after 24 hours})}{\text{Initial wound area}} \times 100 \]

Confocal laser scanning microscopy (CLSM)

Cell morphology was evaluated using CLSM (LSM880, Carl Zeiss, Oberkochen, Germany). All stages of the staining process were carried out at room temperature (23°C) unless otherwise stated. After seeding the cells onto a glass-bottom dish (Matsunami Glass, Tokyo, Japan) and culturing for 24 hours, 1 mL/well of either 25% CSE or 200% HTPE was added. The concentrations of HTPE and CSE were used that had an inhibitory effect on cell migration. The control group was treated with DMEM. After 24 hours of incubation, the medium was aspirated, and the cells were washed with PBS. Subsequently, paraformaldehyde (4%; FUJIFILM Wako Pure Chemical) was added for 30 minutes. After washing with PBS, 0.1% Triton X-100 (Sigma-Aldrich, St. Louis, MO, USA) was added for five minutes. The cells were then washed with PBS and blocked (Block Aid; Invitrogen, Carlsbad, CA) for 30 minutes. The cells were washed with PBS and incubated with anti-α-tubulin mouse IgG (1:100 dilution; Molecular Probes, Eugene, OR, USA) for 60 minutes. Following this, they were washed and incubated with Alexa 568-conjugated goat anti-mouse IgG (1:100 dilution; Molecular Probes), Alexa Fluor 488 phalloidin (Molecular Probes), and 4’,6-diamidino-2-phenylindole (DAPI; 1:100 dilution; Dojindo Laboratories, Kumamoto, Japan) for another 60 minutes. The cells were then washed three times with PBS. Images were captured at magnifications of ×200 and ×630, and a series of Z-stack images were scanned at 2-μm increments using excitation wavelengths of 405, 488, and 568 nm. The maximum projection of each stack was generated using ZEN 2 black software (Carl Zeiss, Oberkochen, Germany).

Quantitative reverse transcription-polymerase chain reaction (qRT-PCR)

HGF-1 cells were seeded (2.5 × 10^4^ cells/well) on 12-well plates (Falcon) and incubated until they reached semi-confluency. Then, DMEM (control), HTPE, or CSE were added, followed by a 24-hour incubation. The total RNA of HGF-1 cultured was isolated with RNeasy® Mini Kit (Qiagen, Valencia, CA, USA) according to the manufacturer's instructions. RNA concentration was determined using a NanoDrop ND-1000 (NanoDrop Technologies, Wilmington, DE, USA). Following quantification, complementary DNA (cDNA) synthesis was performed with the RT2 strand kit RT (Qiagen), following the manufacturer's instructions. qRT-PCR was carried out using StepOnePlus (Applied Biosystems, Carlsbad, CA) with a SYBR Green quanti Nova PCR kit (Qiagen). Measurement of GAPDH served as an internal control. Specific PCR primers for ITGA3, ITGB1, FN1, MMP1, and MMP3 were used (Table 1).

**Table 1:** Primers used for qRT-PCR. qRT-PCR: quantitative reverse transcription-polymerase chain reaction; GAPDH: glyceraldehyde 3-phosphate dehydrogenase; ITGA3: integrin subunit alpha 3; ITGB1: integrin subunit beta 1; FN1: fibronectin 1; MMP-1: matrix metalloproteinase-1; MMP-3: matrix metalloproteinase-3

Gene name	Primer sequences (5'-3')
GAPDH	Forward: GGCTCTCCAGAACATCATCC; Reverse: TTTCTAGACGGCAGGTCAGG
ITGA3	Forward: GGCTGTGTATGGGGAGAAGA; Reverse: TCACCGCGAAGTAGTCACAG
ITAGB1	Forward: GGAGGAATGTTACACGGCTG; Reverse: TTCCTACTGCTGACTTAGGGATC
FN1	Forward: GCCTGGTACAGAATATGTAGTG; Reverse: ATCCCAGCTGATCAGTAGGCTGGTG
MMP-1	Forward: ATGCTGAAACCCTGAAGGTG; Reverse: CTGCTTGACCCTCAGAGACC
MMP-3	Forward: GCAGTTTGCTCAGCCTATCC; Reverse: GAGTGTCGGAGTCCAGCTTTC

The primers were designed using Genbank (https://www.ncbi.nlm.nih.gov/genbank/, accessed on 20 February 2024). Relative gene expression levels were estimated using the 2^−ΔΔCt ^method.

Statistical analyses

A sample size of in vivo experiments was set to 11 per group, assuming that the total difference in bone resorption was 0.6 mm between the periodontitis and the control sides [19], a power of 90%, a standard deviation of 0.4 mm, and a significance level of 0.05%. Considering a 10% dropout, the sample size was set at n=11.

Multiple comparisons for the micro-CT images and qRT-PCR were sought by the Kruskal-Wallis test with Dunn's post hoc test. The nonparametric test was used for these because the normality of data could not be established with the sample size. Comparisons for WST-8 and wound healing assays were made by one-way analysis of variance (ANOVA) with Dunnett's post-test. A software package (Prism 10.4, GraphPad Software, Boston, MA, USA) was used. Statistical significance was established at p < 0.05.

## Results

Nicotine concentration in HTPE and CSE

**Figure 3:**
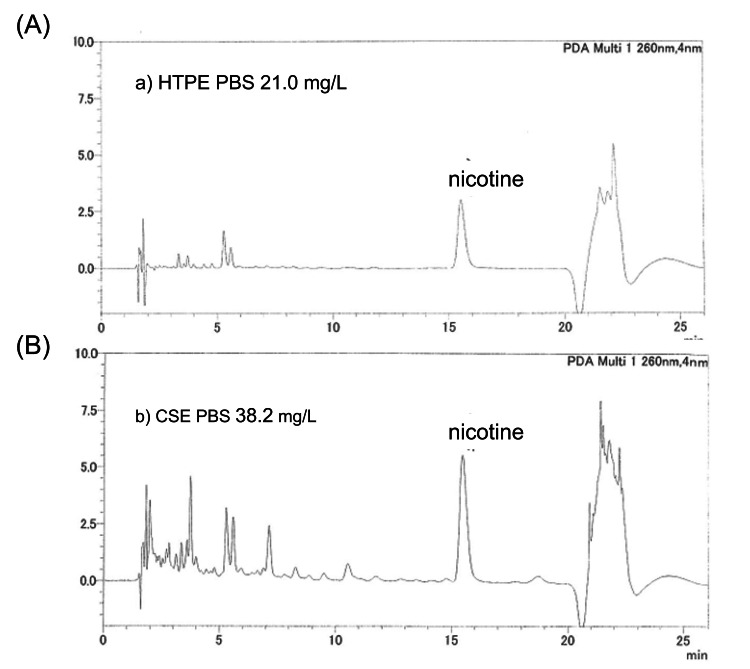
LC/MS analysis of the nicotine concentrations in the samples of HTPE and CSE. (A) Chromatogram of HTPE in PBS. (B) Chromatogram of CSE in PBS. Analyses performed by Shimadzu Techno-Research, Inc., Kyoto, Japan. LC/MC: liquid chromatography-mass spectrometry; HTPE: heated tobacco product extract; CSE: cigarette smoke extract; PBS: phosphate-buffered saline

At the commercial laboratory, liquid chromatography-mass spectrometry (LC/MS) analyses were performed to determine the nicotine concentration in the HTPE and CSE preparations. Representative chromatograms of HTPE and CSE standards in PBS and relative LC/MS spectra are shown in Figure 3.

The retention time (RT) of nicotine peak was 15.5 minutes. The nicotine concentration in CSE preparation was 38.2 mg/L, and that in HTPE was 21.0 mg/L. Based on these, HTPE and CSE were adjusted to a nicotine dose of 0.83 mg/kg for intraperitoneal administration.

HTPE increased alveolar bone resorption and inhibited bone recovery

**Figure 4:**
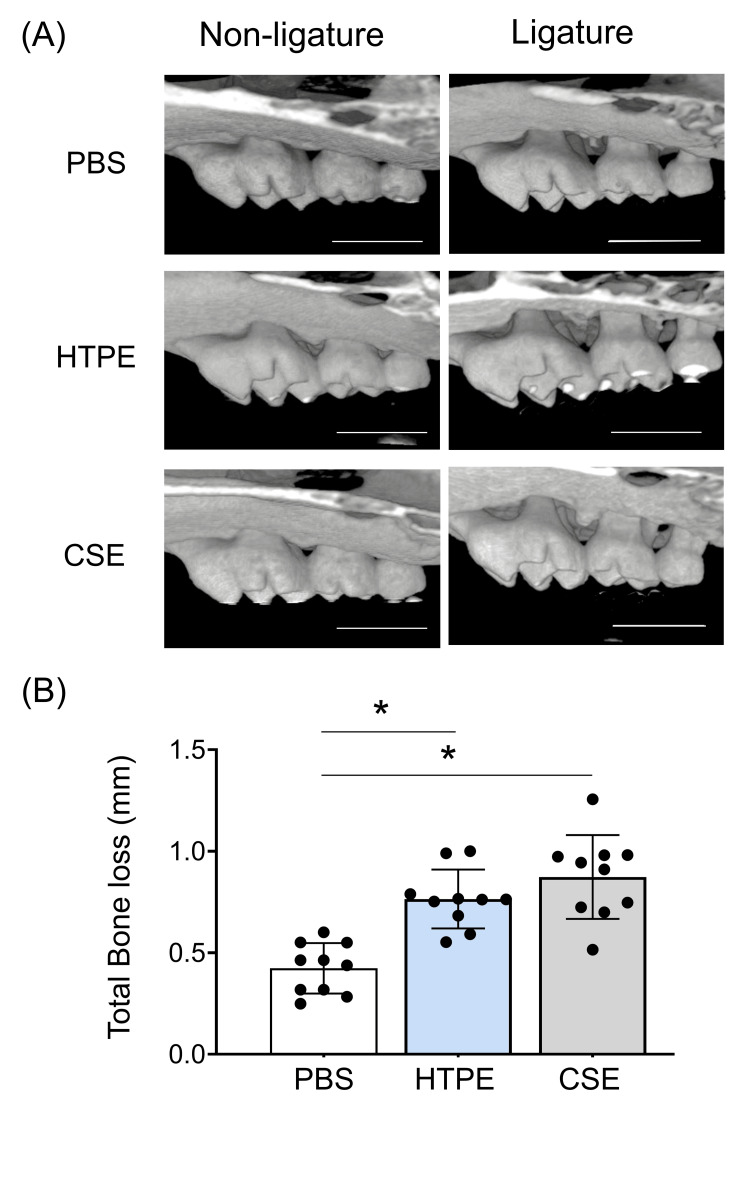
Micro-CT analysis of alveolar bone resorption. (A) Images of the maxillary second molars (palatal side) with or without ligation for seven days (bar = 1000 µm). PBS, HTPE (20 mL/kg), or CSE (20 mL/kg) was given at one, two, and three days before ligature placement. (B) Quantification of alveolar bone resorption on micro-CT in the ligature group. Measurements were taken at six sites along the palatal surface of the maxillae to determine the distance between the cemento-enamel junction (CEJ) and the alveolar bone crest (ABC). The distance at each site was summed up to calculate the total CEJ-ABC distance. The bone resorption in the ligated side in relation to the non-ligated side was calculated as follows: Bone resorption was calculated using the following formula: The difference between the total CEJ-ABC measurements from the ligated side and those from the control side within each mouse. Data show as mean ± SD (n = 10) * p < 0.05 by Kruskal-Wallis test with Dunn’s post hoc test. HTPE: heated tobacco product extract; CSE: cigarette smoke extract; PBS: phosphate-buffered saline

One mouse died during ligation. On day 7, the mouse maxillae were scanned by micro-CT to evaluate the bone resorption. The results of the micro-CT analysis are shown in Figure 4.

The ligation induced rapid and severe bone resorption around the maxillary second molars compared with the control side (Figure 4A). In the HTPE and CSE groups, the levels of alveolar bone resorption were significantly higher than in the control group (p < 0.05) (Figure 4B). There are no significant differences between the HTPE and CSE groups.

**Figure 5:**
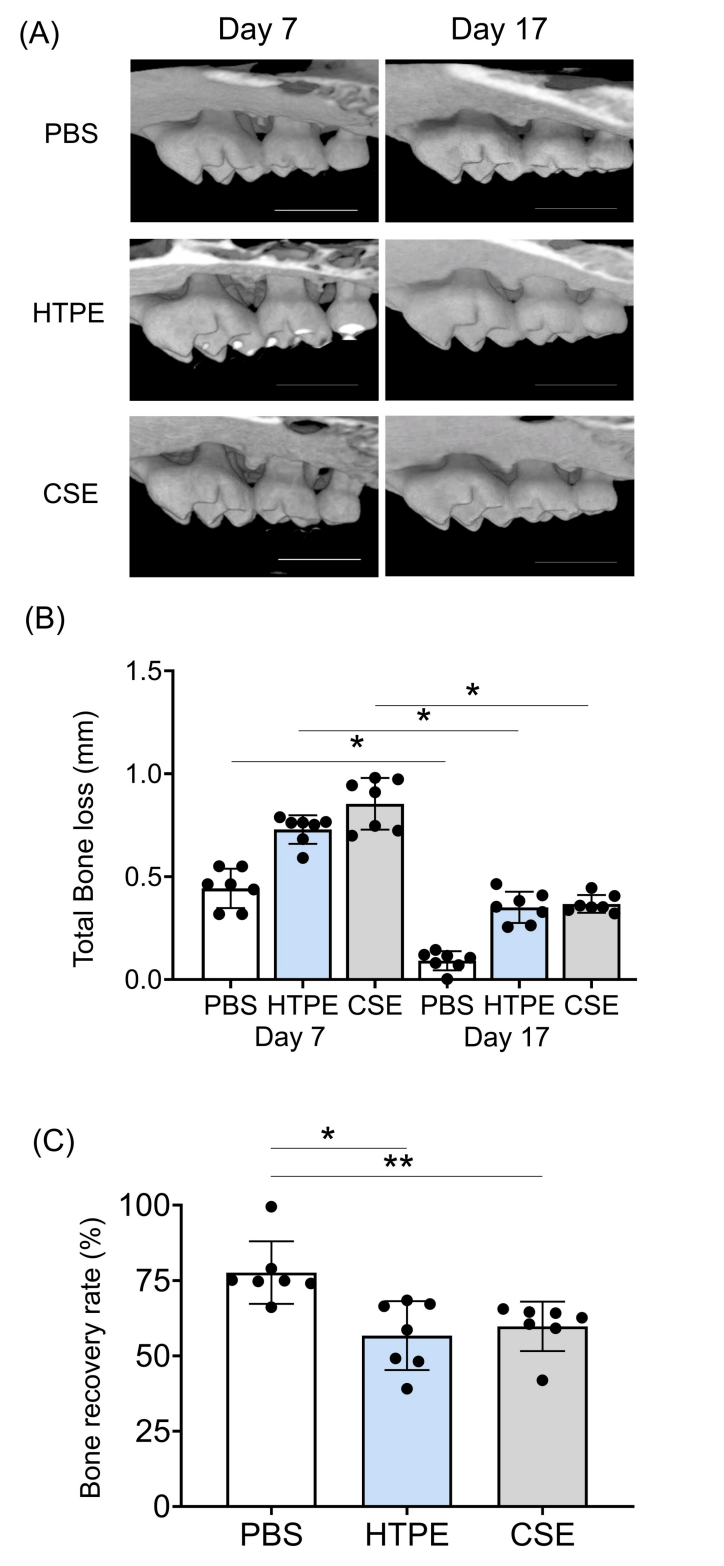
Alveolar bone recovery. (A) Micro-CT images of the maxillary second molars (palatal side) at days 7 and 17 (bar = 1000 μm). (B) Measurement of total bone loss. (C) Rate of alveolar bone recovery = (day 7 total bone loss-day 17 total bone loss)/day 7 total bone lossｘ100. Data are shown as mean ± SD (n=7). * p < 0.05, ** p < 0.01 significant difference values by Kruskal-Wallis test with Dunn's post-test. HTPE: heated tobacco product extract; CSE: cigarette smoke extract; PBS: phosphate-buffered saline

We then assessed the recovery of alveolar bone following ligature removal. Figure 5 shows the results of the micro-CT analysis.

The bone recovery over time was observed in all groups (p < 0.01) (Figures 5A, 5B). At day 17, alveolar bone resorption was significantly reduced compared to day 7 in the HTPE or CSE treatment groups (p < 0.01) (Figure 5B), but the level of recovery was significantly smaller compared with PBS control (p < 0.05) (Figure 5C). These results suggest that HTPE negatively impacts alveolar bone and its healing.

HTPE had no significant effect on cell viability/proliferation

**Figure 6:**
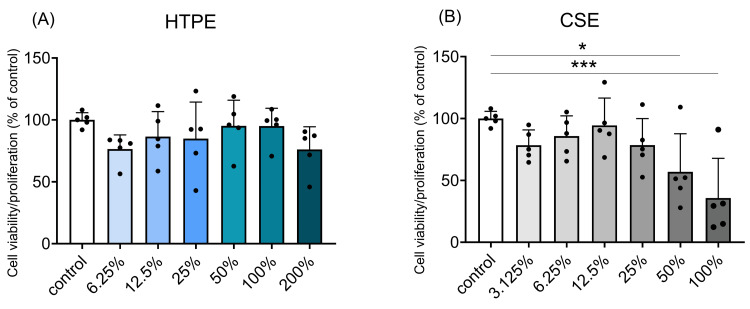
Viability and proliferation of HGF-1. After 24 hours, HGF-1 cells were treated with HTPE (6.25-200%) (A) and CSE (3.125-100%) (B). DMEM was used as a control. After 24 hours, the WST-8 assay was used to assess cell viability/proliferation. Data are expressed as mean ± SD (n = 5). * p < 0.05, *** p < 0.001, significant difference values by one-way analysis of variance (ANOVA) with Dunnett's post hoc test. HGF-1: human gingival fibroblast-1; HTPE: heated tobacco product extract; CSE: cigarette smoke extract; DMEM: Dulbecco's Modified Eagle's Medium

The effects of HTPE or CSE on the viability/proliferation of HGF-1 are shown in Figure 6.

HTPE did not affect the viability/proliferation of HGF-1 at the concentrations tested (Figure 6A). However, CSE (50% and 100%) significantly inhibited the viability/proliferation (p < 0.05 and p < 0.001, respectively) (Figure 6B).

HTPE inhibited cell migration

**Figure 7:**
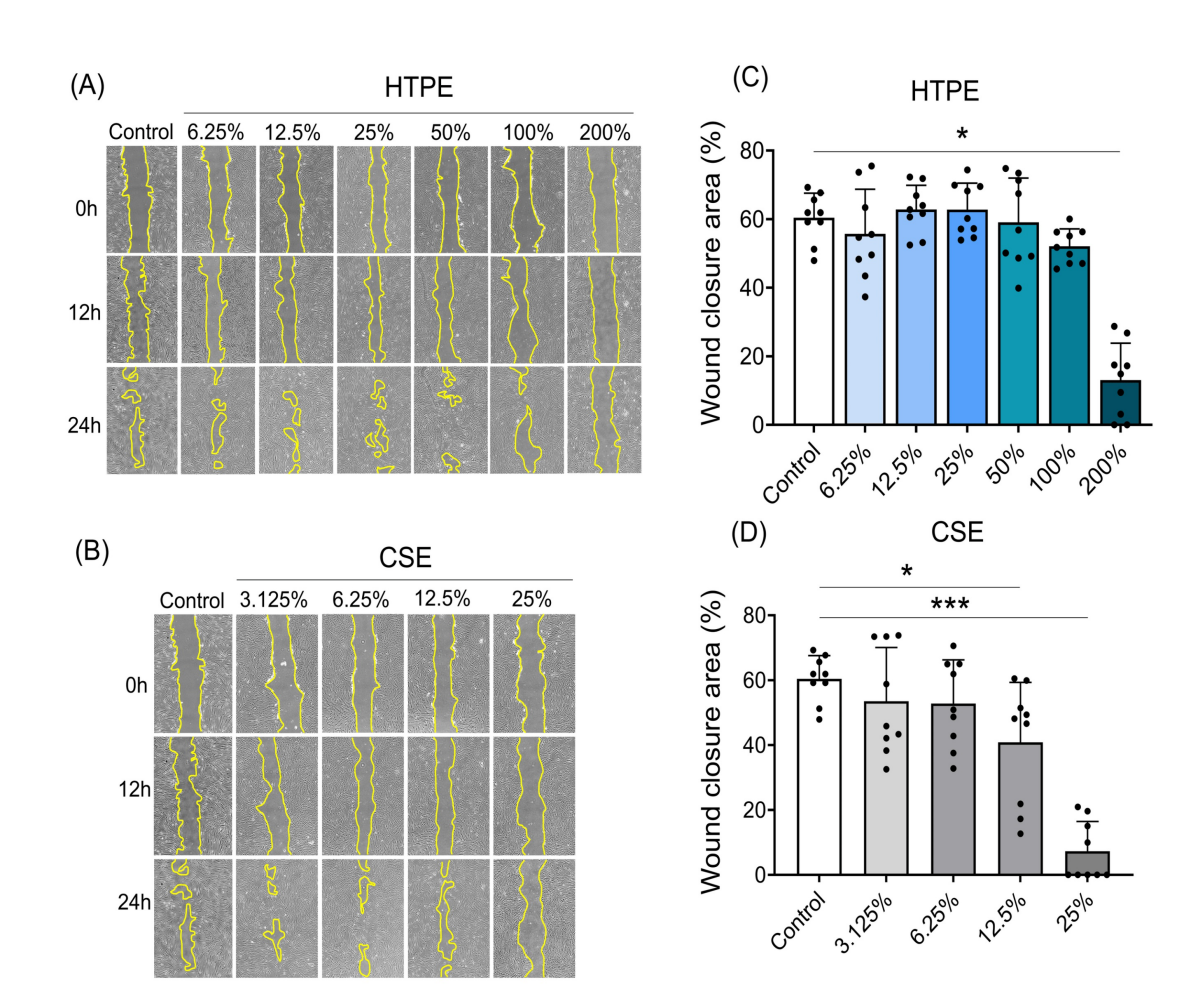
Assessment of cell migration by wound healing assay. Representative microphotographs of the wound closure exposed to HTPE (A) and CSE (B). HGF-1 were cultured to confluence, and multiple artificial wounds were made. Then, various concentrations of HTPE or CSE were added, and the wound area was monitored for up to 24 hours. The rate of wound closure of HGF-1 cells following treatment with HTPE (C) and CSE (D) was obtained using the following formula: \[ \text{Percent wound closure} = \frac{(\text{Initial wound area}) - (\text{Wound area after 24 hours})}{\text{Initial wound area}} \times 100 \] Values are shown as means ± SD (n = 9); * p < 0.05, *** p < 0.001, one-way analysis of variance (ANOVA) with Dunnett's post-test. HTPE: heated tobacco product extract; CSE: cigarette smoke extract; HGF-1: human gingival fibroblast-1

Cell migration was captured using phase-contrast microscopy and evaluated with ImageJ (Figure 7).

HTPE (200%) (Figure 7A) and CSE (12.5% and 25%) (Figure 7B) inhibited migration of HGF-1 compared with the control (Figures 7C, 7D). CSE at concentrations above 50% caused cell detachment, making measurement impossible. In the following experiments, the prepartaions of 200% HTPE and 25% CSE, which inhibited cell migration, were used.

HTPE affected cell cytoskeleton

**Figure 8:**
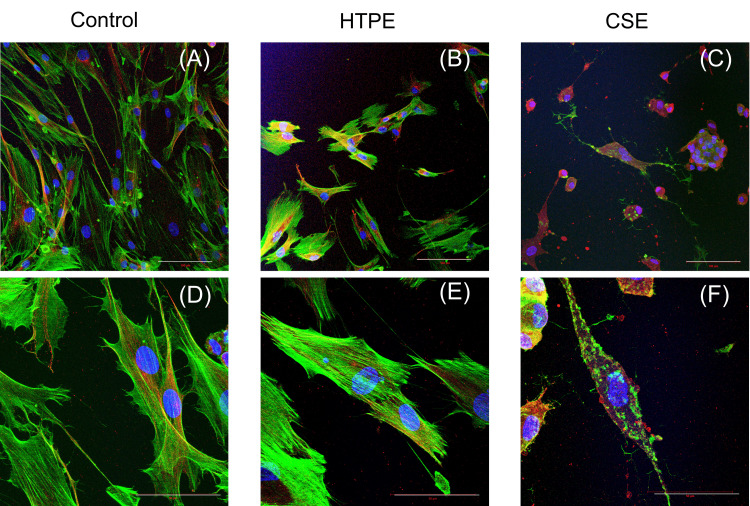
Representative CLSM images of HGF-1 exposed to HTPE and CSE. HGF-1 cells were cultured with medium control (DMEM) (A, D), 200% HTPE (B, E) or 25% CSE (C, F) for 24 hours. Cells were stained for actin (green), tublin (red) and the nucleus (blue). (A-C) Original magnification ×200 (bar =100 μm). (D-F) Original magnification ×630 (bar = 50 μm). HTPE: heated tobacco product extract; CSE: cigarette smoke extract; HGF-1: human gingival fibroblast-1; CLSM: confocal laser scanning microscopy; DMEM: Dulbecco's Modified Eagle's Medium

Cell morphology and cytoskeleton were analyzed using a CLSM (Figure 8).

In the HTPE group, there was a trend toward fewer HGF-1, and the cells exhibited a shorter spindle-shaped morphology compared with the control group (Figures 8B, 8E). Each cell showed fewer filopodia, stronger actin fluorescence intensity, and a greater number of actin filament bundles. In the CSE group, compared with the control group, the cells displayed rounded morphology, with aggregation of actin filaments observed (Figures 8C, 8F).

HTPE promoted gene expression of MMP in HGF-1

**Figure 9:**
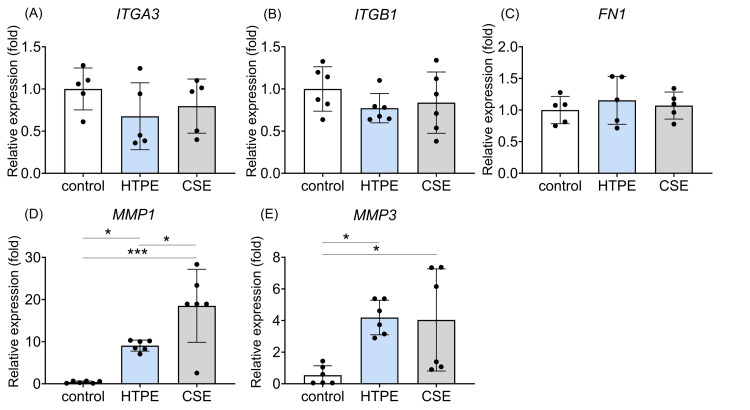
qRT-PCR analysis. HGF-1 cells were treated with culture medium (control), HTPE (200%), or CSE (25%) and further incubated. Gene expressions of integrin subunit alpha 3 (ITGA3) (A), integrin subunit beta 1 (ITGB1) (B), fibronectin (FN1) (C), matrix metalloproteinase-1 (MMP-1) (D) and matrix metalloproteinase-3 (MMP-3) (E) at 24 hours after the treatment are shown. Data are expressed as mean ± SD (n = 5). * p < 0.05, *** p < 0.001 by Kruskal-Wallis test with Dunn’s post hoc test. qRT-PCR: quantitative reverse transcription polymerase chain reaction; HGF-1: human gingival fibroblast-1; HTPE: heated tobacco product extract; CSE: cigarette smoke extract

We next evaluated the expression of genes related to cell migration (Figure 9).

In HGF-1, the treatment with HTPE or CSE yielded no significant change in the expressions of ITGA3, ITGB1, or FN1 compared with the control (Figures 9A-9C). In contrast, HTPE and CSE significantly increased MMP1 and MMP3 expressions (Figures 9D, 9E).

## Discussion

To our knowledge, this study is the first to examine the effects of HTP and CC on periodontal healing by means of in vivo and in vitro approaches. The results suggest that HTPE could exert a negative impact on periodontal tissue.

The methods used to prepare HTPE and CSE vary across studies, making it challenging to compare the results from different studies. While CSE and HTPE are frequently used in research as tobacco smoke/vapor substitutes, the type of tobacco used and the extraction methods are diverse. To minimize quality differences between extracts, we standardized the length of the silicon tubes used and prepared extracts using the puff protocol used in a previous study [16]. Benowitz et al. [23] reported that the blood nicotine concentration in smokers was 2.0 to 3.7 × 10^-2^ µg/mL. Hoffmann and Adams [21] reported a urinary nicotine concentration of 2.0 × 10^-1 ^µg/mL and a salivary concentration of 1.56 × 10^3^ µg/mL in smokers. In a previous study from our group using gingival epithelial cells, a nicotine concentration of 5.0 × 10 µg/mL was used [8]. A study using gingival fibroblasts employed 8.1 × 10^2^ µg/mL [24], and those using periodontal ligament fibroblasts utilized 2.5 × 10^2^ µg/mL [25]. Additionally, a protocol for intraperitoneal administration using nicotine (16 µg per 20 g of mouse weight) was used in a periodontitis mouse model [18]. In the present study, the HTPE contained 21.0 μg/mL of nicotine, while the CSE contained 38.2 μg/mL. The range of nicotine concentrations used in the present experiment was based on these results: 1.23-42.0 mg/L for HTPE (6.25-200%) and 1.19-38.2 mg/L for CSE (3.125-100%). In the present study, we wished to focus on the effects of HTPE on periodontal tissue after entering the systemic circulation. Therefore, we used the same methods as in the previous study [18] to examine the effects.

Chemicals in cigarette smoke, including nicotine, are absorbed into the bloodstream and subsequently disseminated throughout the body's circulatory system [4]. In periodontal tissues, these components may also contribute to the progression of periodontal disease via systemic circulation. Consequently, to evaluate the impact of HTPE and CSE on periodontal tissues through systemic circulation, we delivered these extracts via intraperitoneal injection. The intraperitoneal dosage was determined based on the nicotine amount. There is a view that HTPE contains fewer harmful substances, including nicotine, compared to CSE. Nonetheless, a previous study showed that the overall nicotine concentrations of HTPE and CSE were approximately equal [26]. Therefore, in our in vivo experiments, the dosage was adjusted so that the nicotine concentrations in the HTPE and CSE were equivalent.

A previous study has highlighted the destructive effects of CSE on periodontal tissues [18]. In our in vivo experiment, intraperitoneal administration of HTPE and CSE induced similar levels of alveolar bone resorption at the tested concentrations. Moreover, after ligature removal, the extent of alveolar bone recovery was similar in the HTPE and CSE groups. Our in vitro data demonstrated that the exposure to HTPE upregulated MMP-1 and MMP-3 expressions in HGF. MMP is implicated in the remodeling of periodontal tissues, wound healing, and inflammatory responses. In patients with chronic periodontitis who smoke, MMP expression and activity have been reported to be markedly increased [27]. Excessive MMP-1 expression contributes to alveolar bone resorption, exacerbating the destruction of periodontal tissues [28]. Therefore, HTP use may also impair periodontal wound healing, and the effects on ECM may be one of the underlying mechanisms. The effects of HTPE on periodontal ligament stem cells, their osteoblastic differentiation, and inflammatory responses need to be investigated to fully explain the in vivo observations of alveolar bone changes.

Cell migration occurs by extending filopodia protrusions in the direction of movement. This process stabilizes through adhesion to the extracellular matrix (ECM) or adjacent cells via transmembrane receptors linked to the actin cytoskeleton. Efficient cell migration requires a dynamic balance between actin assembly and disassembly [29]. In the present study, HTPE enhanced the expression of actin filaments in HGF-1, while the cells presented rounded morphology with actin aggregation. This finding may indicate that CSE exerts more pronounced structural damage than HTPE. The reduced cell migration, coupled with increased actin bundling, suggests that HTPE affects cellular mechanics and motility apparatus. Our observation that gene expressions of integrins and fibronectin remained unchanged suggests that HTPE does not affect cell-ECM adhesion at the transcriptional level. A previous study reported that CSE increased the expression of MMP-1 and MMP-3 in human fibroblasts, promoting collagen degradation and inhibiting collagen biosynthesis [30]. In the present study, the increased MMP gene expression could be interpreted as a cellular attempt to compensate for reduced mobility by enhancing ECM degradation, though this was not sufficient to overcome the mechanical constraints imposed by the stabilized actin cytoskeleton. Based on these results, it is suggested that the HTPE has an inhibitory effect on periodontal tissue healing, possibly via ECM remodeling. Further experiments, including the examination of focal adhesion dynamics and key signaling molecules, are necessary to explore the more detailed mechanisms.

There are several limitations to this study. The diversity of human smoking behaviors presents inherent challenges for both in vitro and in vivo replication. While we utilized HTPE concentrations within previously reported ranges, our administration method involved acute systemic rather than chronic exposure. Thus, our experimental design may not fully capture the effects of actual vapor/smoke exposure on the human oral cavity, as tobacco usage typically involves multiple exposures throughout the day over extended periods. A more physiologically relevant exposure model, such as direct application to the oral cavity or aerosol inhalation, should be tested across various temporal exposure patterns and consumption behaviors. For in vitro experiments, a cell line was used, and the observed effects of HTPE on cell behaviors or response patterns may differ compared with primary cells. Furthermore, HTPE contains additional chemicals of concern not typically measured in CC smoke. The role of these chemicals in the observed effects of HTPE remains to be elucidated. Despite these limitations, our results contribute to a better understanding of the impact of HTP use on periodontal health.

## Conclusions

Our study demonstrates that HTPE administration exacerbated alveolar bone resorption and impaired bone recovery in a mouse model of periodontitis. In vitro, HTPE significantly inhibited gingival fibroblast migration, in part through modulation of MMP-related gene expression. While the magnitude differs from that of CSE, these results suggest that HTPE at the given concentrations adversely affects periodontal tissue and its healing processes.
